# Hetero-Type Benzannulation Leading to Substituted Benzothio-Phenes

**DOI:** 10.3390/molecules26227008

**Published:** 2021-11-19

**Authors:** Taro Kono, Ryosuke Sasaki, Hideki Goto, Masatoshi Kakuno, Yoo Tanabe

**Affiliations:** Department of Chemistry, School of Science and Technology, Kwansei Gakuin University, 2-1 Gakuen, Sanda 669-1337, Japan; eui39247@kwansei.ac.jp (T.K.); dhc19718@kwansei.ac.jp (R.S.); edj32736@kwansei.ac.jp (H.G.); dbk33447@kwansei.ac.jp (M.K.)

**Keywords:** thiophene, benzothiophene, benzannulation, gem-dichlorocyclopropane, Suzuki–Miyaura cross-coupling, hydroxylation, borylation, titanium tetrachloride, tin tetrachloride

## Abstract

TiCl_4_ (or SnCl_4_)-promoted hetero-type benzannulation reactions using various (2,2-dichlorocyclopropyl)(thiophen-2-yl)methanols proceeded smoothly to produce uniquely substituted 4-chlorobenzothiophenes (five examples). The present approach involves the first distinctive thiophene formation from thiophene cores, in contrast to traditional methods of thiophene formation from benzene cores. The stereocongested (less reactive) Cl position in the obtained 4-chlorobenzothiophenes functioned successfully as the partners of three cross-coupling reactions: (i) a Suzuki–Miyaura cross-couplings using Pd(OAc)_2_/SPhos/K_3_PO_4_ catalysis (seven examples; 63–91%), (ii) a hydroxylation using KOH/Pd(dba)_2_/tBu-XPhos catalysis (85%), and (iii) a borylation using a B_2_(pin)_2_/Pd(dba)_2_/XPhos/NaOAc catalysis-provided 4-(pin)B-benzothiophene (58%).

## 1. Introduction

Benzothiophenes are well-recognized, basic sulfur-containing heterocycles as thiophene benzologues, and are utilized as key pharmacophores [1,2]. Raloxifene (an anti-cancer drug) [3], sertaconazole (an anti-fungal drug) [4], benocyclidine (a psychoactive recreational drug) [5], zileuton (a lipoxygenase inhibitor) [6], etc., are representative examples.

**Scheme 1:**
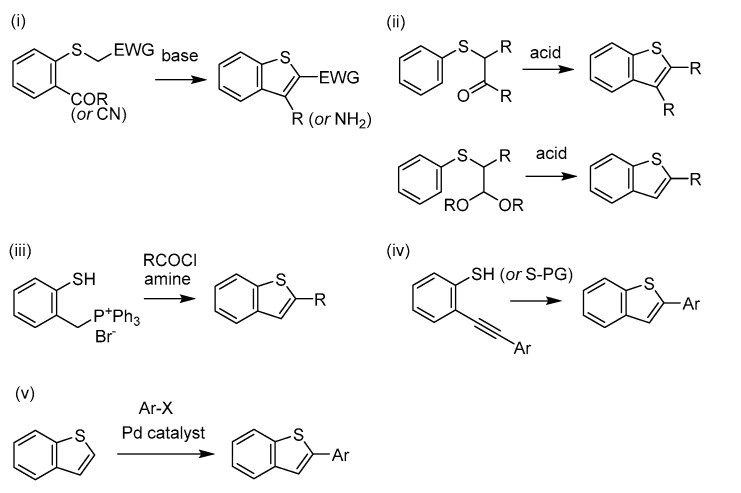
Representative synthetic methods for benzothiophenes.

Therefore, a number of syntheses have been developed to date [1,2]. Representative methods for the construction of simple, unsubstituted benzothiophenes are categorized into several approaches (Scheme 1): (i) Hinsberg-type annulations [7,8,9], (ii) Friedel–Crafts type annulations [10,11,12,13], (iii) Wittig-type condensations of phosphonium salts [14,15], (iv) Metal-catalyzed thiolation annulations [16,17,18], (v) Pd-catalyzed C-H arylations [19], and others [20,21,22,23,24]. 

**Scheme 2:**
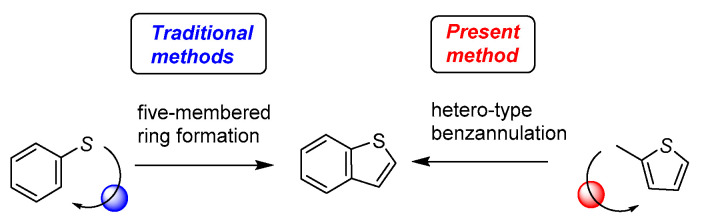
Annulation synthetic methods for benzothiophenes.

**Scheme 3:**
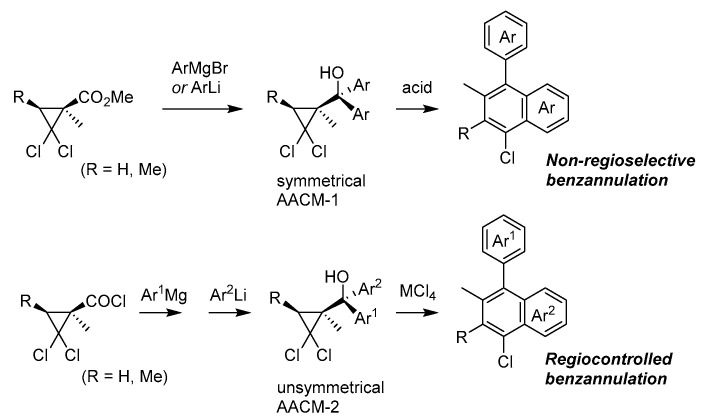
Two types of benzannulation for naphthalene formation.

These traditional syntheses consistently utilize thiophene formations from the benzene cores. Taking this background into account, we envisaged a unique synthetic approach for the construction of benzothiophenes from counter thiophene cores, which is one type of benzannulation strategy (Scheme 2). Our group previously investigated primary non-regioselective [25] and secondary regiocontrolled [26,27] benzannulation methodologies; symmetrical (diaryl)(2,2-dichloro-1-methylcyclopropyl)methanols (AACM-1) and non-symmetrical and stereodefined (aryl-1)(aryl-2)(2,2-dichloro-1-methylcyclopropyl)methanols (AACM-2) underwent the reactions to produce distinct 1-aryl-4-chloronaphthalene families bearing various substituents (Scheme 3). An ipso-variant of the regiocontrolled benzannulation for synthesizing uniquely substituted α-arylnaphthalenes and its application to the total synthesis of chaihunaphthone was also disclosed [27]. Recently, Anilkumar and co-workers provided a comprehensive review of the synthetic application of 1,1-dihalocyclopropanes [28].

## 2. Results and Discussion

**Scheme 4:**
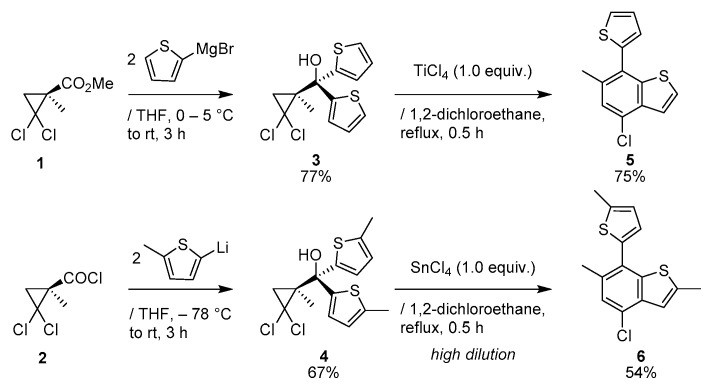
Benzothiophene formations by a hetero-type benzannulation strategy.

Our initial attempts were guided by the reaction using (2,2-dichloro-1-methylcyclopropyl)di(thiophen-2-yl)methanols **3** and **4** (Scheme 4). Alcohol **3** was prepared from commercially and/or readily available methyl 2,2-dichloro-1-methylcyclopropanecarboxylate (**1**) with 2-thienylmagnesium bromide, whereas the reaction between the lithium salt of 2-methylthiophene and acid chloride **2** was applied for the preparation of **3** due to the less reactivity of the lithium salt of 2-methylthiophene.

The TiCl_4_-promoted hetero-type benzannulation using alcohol **3** proceeded successfully, affording the desired 4-chloro-6-methyl-7-(thiophen-2-yl)benzothiophene (**5**) in 75% yield. Although the reaction of alcohol **4** using TiCl_4_ unfortunately resulted in complex mixtures, a substitution with SnCl_4_ successfully afforded the corresponding benzothiophene **6** in 54% yield. 

**Scheme 5:**
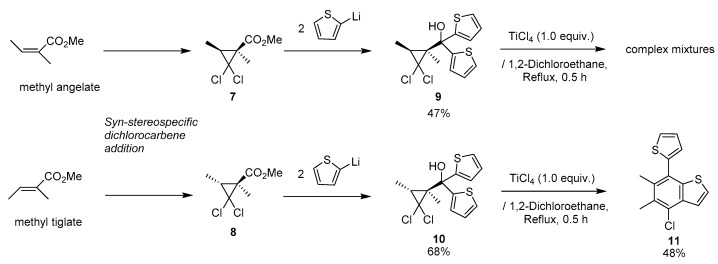
Stereochemical features of the hetero-type benzannulation.

Hetero-type benzannulation using diastereoisomeric (2,2-dichloro-1,3-dimethylcyclopropyl)di(thiophen-2-yl)methanols **9** and **10** afforded intriguing results (Scheme 5). Alcohol **9** was prepared from methyl angelate by the addition of stereospecific *syn*-dichlorocarbene and the subsequent addition of the two molar 1-lithiated thiophene through methyl ester **7**. In a similar procedure, isomeric methyl tiglate was converted to alcohol **10** through methyl ester **8**. The identical TiCl_4_-mediated and SnCl_4_-mediated reactions using **9**, however, yielded only complex mixtures. To our delight, **10** successfully underwent hetero-benzannulation to afford **11** in 48% yield. This outcome is in clear contrast to the benzannulations for naphthalene formation, wherein methyl angelate was employed as a starting compound [9,10]. The reason for the contrast switching results using diastereomeric substrates is not clear at present. 

**Scheme 6:**
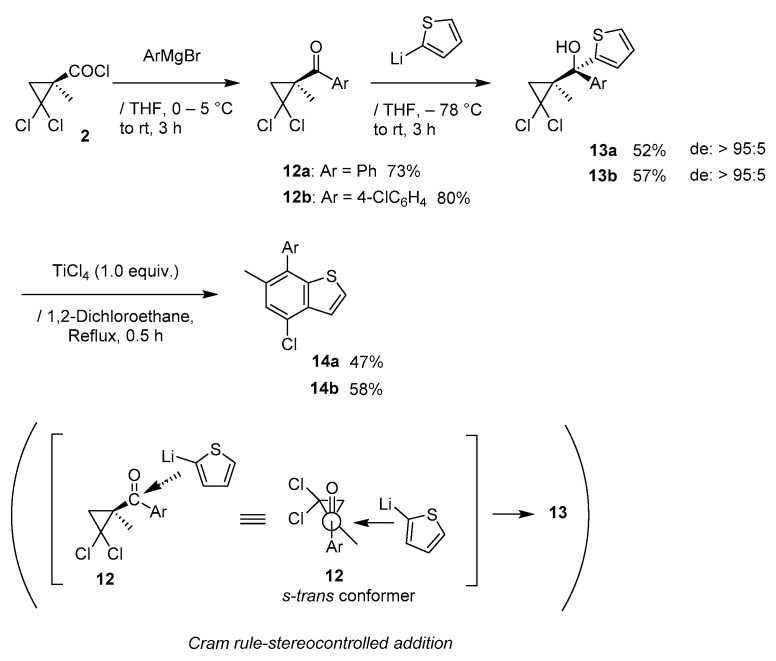
Regiocontrolled hetero-type benzannulation.

Next, the regiocontrol aspect of the present hetero-benzannulation is discussed (Scheme 6). Following the reported procedure for the preparation of AACM-2 (Scheme 3) [10], the sequential introduction of Ar groups and a 1-thienyl group to acid chloride **2** provided stereodefined alcohols **13a** and **13b** in good yield with excellent stereoselectivity through ketones **12a [26]** and **12b**, respectively. The stereochemical course of the diastereoselective addition accounts for the reported mechanistic speculation based on the Cram rule [25,26,27]; the thienyl anion attacks the less hindered side of the more stable s-trans conformer of ketones **12** to afford stereodefined alcohols **13** with >95:5 de.

The distinctive hetero-type benzannulation procedure using **13a** and **13b** successfully produced 6-arylbenzothiophenes **14a** and **14b** in 47% and 58% yields, respectively, with high regiocontrol (Electronic Supporting Information of Free Energy Calculations: see SI).

**Figure 1:**
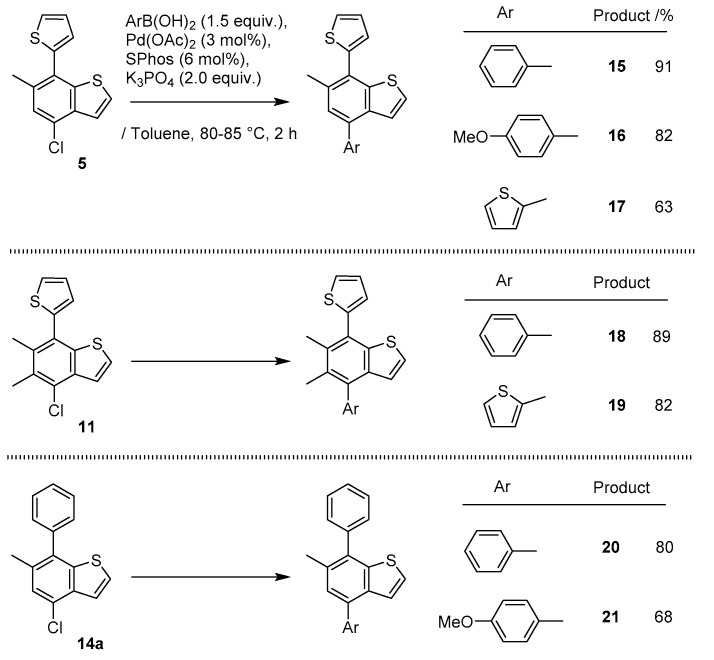
Suzuki–Miyaura cross-coupling of 4-chlorobenzothiophenes.

With these successful results in hand, we investigated the functionalization of the obtained benzothiophenes **5**, **11**, and **14a** to demonstrate the utility for synthesizing seven 4-aryl-substituted benzothiophene derivatives **15**–**21**. As depicted in Figure 1, the Suzuki–Miyaura cross-couplings proceeded smoothly at the congested (less reactive) 4-Cl-position using Pd(OAc)_2_/SPhos/K_3_PO_4_ catalysis to produce a variety of uniquely substituted benzothiophenes **15**‒**21** in good to excellent yield. The use of K_3_PO_4_ was superior to that of K_2_CO_3_ (70%) and *i*-Pr_2_NEt (65%). 

**Scheme 7:**
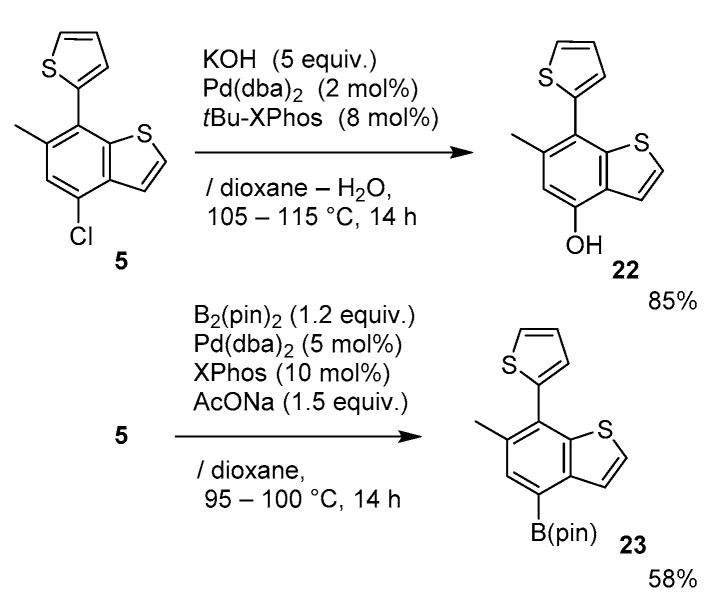
Two types of cross-couplings leading to 4-heteroatom-substituted benzothiophenes.

As a further distinctive extension, a couple of heteroatom groups [OH- and (pin)B-] were successfully introduced into benzothiophene **5** using recently developed cross-coupling methods; KOH/Pd(dba)_2_/tBu-XPhos catalysis [29] provided 4-hydroxybenzothiophene **22**, whereas B_2_(pin)_2_/Pd(dba)_2_/XPhos/NaOAc catalysis [30] provided 4-(pin)B-benzothiophene **23** (Scheme 7). 

## 3. Materials and Methods

(S*)-(2,2-Dichloro-1-methylcyclopropyl)di(thiophen-2-yl)methanol (**3**) 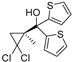


2-Bromothiophene (2.45 g, 15.0 mmol) was added to a stirred suspension of Mg (365 mg, 15.0 mmol) in THF (15 mL) at 20–25 °C under an Ar atmosphere, and the mixture was stirred at the same temperature for 1 h. Methyl (1S*)-2,2-dichloro-1-methylcyclopropanecarboxylate (commercially available or prepared by the reported method [9]) (**1**; 549 mg, 3.0 mmol) in THF (3.0 mL) was added to the mixture at 0–5 °C, and was stirred at 20–25 °C for 3 h. Sat. NH_4_Cl aqueous solution was added to the mixture, which was extracted twice with AcOEt. The combined organic phase was washed with water, brine, dried (Na_2_SO_4_) and concentrated. The obtained crude oil was purified by SiO_2_ column chromatography (hexane/AcOEt = 50:1) to give the desired product **3** (739 mg, 77%). 

Pale yellow oil; Rf = 0.49 (hexane/AcOEt = 10:1); ^1^H NMR (500 MHz, CDCl_3_): δ = 1.35 (d, 1H, *J* = 7.5 Hz), 1.36 (s, 3H), 2.48 (d, 1H, *J* = 7.5 Hz), 3.24 (s, 1H), 6.72–6.74 (m, 1H), 6.88–6.91 (m, 1H), 7.06–7.09 (m, 1H), 7.29–7.32 (m, 1H), 7.33–7.35 (m, 1H), 7.40–7.42 (m, 1H); ^13^C NMR (125 MHz, CDCl_3_): δ = 22.3, 28.7, 39.1, 67.4, 77.2, 125.6, 125.9, 126.2, 126.6, 126.9, 127.3, 146.7, 149.8; IR (neat): ν_max_ = 3545, 3103, 3000, 1663, 1319, 1020, 667 cm^−1^; HRMS (DART): *m*/*z* calcd for C_13_H_12_Cl_2_OS_2_ [*M* − OH]^+^ 300.9679; found: 300.9674.

(S*)-(2,2-Dichloro-1-methylcyclopropyl)bis(5-methylthiophen-2-yl)methanol (**4**) 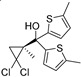


*n*BuLi (1.57 M in hexane, 5.73 mL, 9.0 mmol) was added to a stirred solution of 2-methylthiophene (883 mg, 9.0 mmol) in THF (6.75 mL) at −78 °C under an Ar atmosphere, and the mixture was stirred at the same temperature for 1 h. 2,2-Dichloro-1-methylcyclopropanecarbonyl chloride [9] (**2**; 562 mg, 3.0 mmol) in THF (2.25 mL) was added to the mixture at the same temperature, and gradually warmed up to 20–25 °C for 3 h. Sat. NH_4_Cl aqueous solution was added to the mixture, which was extracted twice with AcOEt. The combined organic phase was washed with water, brine, dried (Na_2_SO_4_) and concentrated. The obtained crude oil was purified by SiO_2_ column chromatography (hexane/AcOEt = 30:1) to give the desired product **4** (571 mg, 67%).

Pale yellow oil; Rf = 0.65 (hexane/AcOEt = 10:1); ^1^H NMR (500 MHz, CDCl_3_): δ = 1.31 (d, 1H, *J* = 7.5 Hz), 1.36 (s, 3H), 2.43 (d, 1H, *J* = 7.5 Hz), 2.45 (s, 3H), 2.51 (s, 3H), 3.10 (s, 1H), 6.52–6.56 (m, 2H), 6.68–6.71 (m, 1H), 7.08–7.09 (m, 1H); ^13^C NMR (125 MHz, CDCl_3_): δ = 15.3, 15.4, 22.6, 28.7, 38.8, 67.4, 77.2, 123.7, 124.3, 126.7, 127.2, 140.4, 141.1, 143.9, 147.3; IR (neat): ν_max_ = 3555, 2920, 1449, 1231, 1018, 907 cm^−1^; HRMS (DART): *m*/*z* calcd for C_15_H_16_Cl_2_OS_2_ [*M* − OH]^+^ 328.9992; found: 328.9965.

((1S*,3S*)-2,2-Dichloro-1, 3-dimethylcyclopropyl)di(thiophen-2-yl)methanol (**9**) 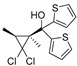


Following the procedure for the preparation of **4**, the reaction using methyl (1S*,3S*)-2,2-dichloro-1,3-dimethylcyclopropane-1-carboxylate [9] **7** (591 mg, 3.0 mmol) derived from methyl angelate, *n*BuLi (1.55 M in hexane, 9.68 mL, 15.0 mmol), and thiophene (1.26 g, 15.0 mmol) in THF (18 mL) gave the crude oil, which was purified by SiO_2_ column chromatography (hexane/AcOEt = 30:1) to give the desired product **9** (468 mg, 47%).

Pale yellow oil; Rf = 0.35 (hexane/AcOEt = 10:1); ^1^H NMR (500 MHz, CDCl_3_): δ = 1.35 (s, 3H), 1.59 (q, 1H, *J* = 6.9 Hz), 1.73 (d, 3H, *J* = 6.9 Hz), 3.22 (s, 1H), 6.75–6.77 (m, 1H), 6.89–6.91 (m, 1H), 7.04–7.07 (m, 1H), 7.31–7.34 (m, 2H), 7.38–7.40 (m, 1H); ^13^C NMR (125 MHz, CDCl_3_): δ = 10.7, 26.3, 37.1, 39.5, 73.0, 80.0, 125.6, 126.0, 126.2, 126.4, 127.0, 127.4, 148.5, 150.0; IR (neat): ν_max_ = 3557, 3107, 2932, 2361, 1450, 1026, 700 cm^−1^; HRMS (DART): *m*/*z* calcd for C_14_H_14_C_l2_OS_2_ [*M* − OH]^+^ 314.9836; found: 314.9814.

((1S*,3R*)-2,2-Dichloro-1, 3-dimethylcyclopropyl)di(thiophen-2-yl)methanol (**10**) 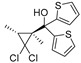


Following the procedure for the preparation of **4**, the reaction using methyl (1S*,3R*)-2,2-dichloro-1,3-dimethylcyclopropane-1-carboxylate [9] **8** (985 mg, 5.0 mmol) derived from methyl tiglate, *n*BuLi (1.57 M in hexane, 15.9 mL, 25.0 mmol), and thiophene (2.10 g, 25.0 mmol) in THF (30 mL) gave the crude oil, which was purified by SiO_2_ column chromatography (hexane/AcOEt = 30:1) to give the desired product **10** (1.13 g, 68%).

Pale yellow oil; Rf = 0.47 (hexane/AcOEt = 10:1); ^1^H NMR (500 MHz, CDCl_3_): δ = 1.13 (s, 3H), 1.18 (d, 3H, *J* = 6.9 Hz), 2.61 (q, 1H, *J* = 6.9 Hz), 3.25 (s, 1H), 6.70–6.72 (m, 1H), 6.85–6.88 (m, 1H), 7.05–7.08 (m, 1H), 7.28–7.33 (m, 2H), 7.39–7.42 (m, 1H); ^13^C NMR (125 MHz, CDCl_3_): δ = 9.0, 16.5, 27.4, 40.1, 71.7, 77.7, 125.5, 126.0, 126.2, 126.5, 126.8, 127.3, 146.8, 149.9; IR (neat): ν_max_ = 3547, 3105, 2934, 2361, 1236, 835, 700 cm^−1^; HRMS (DART): *m*/*z* calcd for C_14_H_14_Cl_2_OS_2_ [*M* − OH]^+^ 314.9836; found: 314.9833.

(S*)-[(S*)-2,2-Dichloro-1-methylcyclopropyl(phenyl)]methanone [9] (**12a**)

(S*)-(4-Chlorophenyl)(2,2-dichloro-1-methylcyclopropyl)methanone (**12b)** 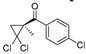


1-Bromo-4-chlorobenzene (1.15 g, 6.0 mmol) was added to a stirred suspension of Mg (146 mg, 6.0 mmol) in THF (5 mL) at 20–25 °C under Ar atmosphere, and the mixture was stirred at the same temperature for 1 h. Acid chloride **2** (937 mg, 5.0 mmol) in THF (5.0 mL) was added to the mixture at 0–5 °C, which was stirred at 20–25 °C for 3 h. Sat.NH_4_Cl aqueous solution was added to the mixture, which was extracted twice with AcOEt. The combined organic phase was washed with water, brine, dried (Na_2_SO_4_) and concentrated. The obtained crude oil was purified by SiO_2_ column chromatography (hexane/AcOEt = 30:1) to give the desired product **12b** (1.06 g, 80%). 

Colorless oil; Rf = 0.63 (hexane/AcOEt = 10:1); ^1^H NMR (500 MHz, CDCl_3_): δ = 1.50 (d, 1H, *J* = 7.5 Hz), 1.63 (s, 3H), 2.29 (d, 1H, *J* = 7.5 Hz), 7.49–7.55 (m, 2H), 7.87–7.92 (m, 2H); ^13^C NMR (125 MHz, CDCl_3_): δ = 20.6, 30.0, 39.6, 62.2, 129.1 (2C), 131.0 (2C), 132.8, 139.9, 194.3; IR (neat): ν_max_ = 3090, 2936, 1684, 1587, 1091, 986, 773 cm^−1^; HRMS (DART): *m*/*z* calcd for C_11_H_9_Cl_3_O [*M* + H]^+^ 262.9797; found: 262.9790.

(S*)-[(S*)-2,2-Dichloro-1-methylcyclopropyl)(phenyl)(thiophen-2-yl)]methanol (**13a**) 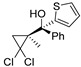


*n*BuLi (1.55 M in hexane, 6.45 mL, 10.0 mmol) was added to a stirred solution of thiophen (841 mg, 10.0 mmol) in THF (7.5 mL) at −78 °C under an Ar atmosphere, and the mixture was stirred at the same temperature for 1 h. Ketone **12a** (1.15 g, 5.0 mmol) in THF (2.5 mL) was added to the mixture at the same temperature, and gradually warmed up to 20–25 °C for 3 h. Sat. NH_4_Cl aqueous solution was added to the mixture, which was extracted twice with AcOEt. The combined organic phase was washed with water, brine, dried (Na_2_SO_4_) and concentrated. The obtained crude oil was purified by SiO_2_ column chromatography (hexane/AcOEt = 50:1) to give the desired product **13a** (813 mg, 52%).

Pale yellow oil; Rf = 0.40 (hexane/AcOEt = 30:1); ^1^H NMR (500 MHz, CDCl_3_): δ = 1.29 (s, 3H), 1.32 (d, 1H, *J* = 7.5 Hz), 2.48 (d, 1H, *J* = 7.5 Hz), 2.96 (s, 1H), 6.44–6.46 (m, 1H), 6.85–6.88 (m, 1H), 7.28–7.31 (m, 1H), 7.37–7.48 (m, 3H), 7.62–7.66 (m, 2H); ^13^C NMR (125 MHz, CDCl_3_): δ = 23.0, 28.0, 37.3, 68.0, 79.7, 125.5, 125.6, 126.7, 128.2 (2C), 128.5, 128.9 (2C), 142.0, 150.9; IR (neat): ν_max_ = 3563, 3296, 3088, 2941, 1022, 762, 700 cm^−1^; HRMS (DART): *m*/*z* calcd for C_15_H_14_Cl_2_OS [*M* − OH]^+^ 295.0115; found: 295.0109.

(S*)-(4-Chlorophenyl)((S*)-2,2-dichloro-1-methylcyclopropyl)(thiophen-2-yl)methanol (**13b**) 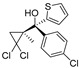


Following the procedure for the preparation of **2a**, the reaction using ketone **12b** (1.05 g, 4.0 mmol), *n*BuLi (1.55 M in hexane, 5.16 mL, 8.0 mmol), and thiophene (676 mg, 8.0 mmol) in the THF (8.0 mL) gave the crude oil, which was purified by SiO_2_ column chromatography (hexane/AcOEt = 50:1) to give the desired product **13b** (766 mg, 57%).

Pale yellow oil; Rf = 0.53 (hexane/AcOEt = 10:1); ^1^H NMR (500 MHz, CDCl_3_): δ = 1.26 (s, 3H), 1.33 (d, 1H, *J* = 7.5 Hz), 2.64 (d, 1H, *J* = 7.5 Hz), 2.97 (s, 1H), 6.43–6.45 (m, 1H), 6.86–6.88 (m, 1H), 7.30–7.31 (m, 1H), 7.40–7.44 (m, 2H), 7.55–7.59 (m, 2H); ^13^C NMR (125 MHz, CDCl_3_): δ = 23.0, 28.0, 37.6, 67.7, 79.3, 125.6, 125.7, 126.7, 128.4 (2C), 130.4 (2C), 134.4, 140.6, 150.4; IR (neat): ν_max_ = 3555, 3075, 3001, 1491, 1094, 1024, 704 cm^−1^; HRMS (DART): *m*/*z* calcd for C_15_H_13_Cl_3_OS [*M − OH*]^+^ 328.9725; found: s328.9733.

4-Chloro-6-methyl-7-(thiophen-2-yl)benzo[b]thiophene (**5**) 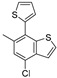


TiCl_4_ (1.0 M in 1,2-dichloroethane, 4.1 mL, 4.1 mmol) was added to a solution of alcohol **3** (1.32 g, 4.1 mmol) in 1,2-dichloroethane (83 mL) at 80 °C under an Ar atmosphere, and the mixture was stirred at the same temperature for 0.5 h. After cooling down to room temperature, sat. NaHCO_3_ aqueous solution was added to the mixture, which was extracted twice with AcOEt. The combined organic phase was washed with water, brine, dried (Na_2_SO_4_) and concentrated. The obtained crude oil was purified by SiO_2_ column chromatography (hexane) to give the desired product **5** (822 mg, 75%).

Colorless crystals; Rf = 0.34(hexane); mp 67–68 °C; ^1^H NMR (500 MHz, CDCl_3_): δ = 2.35 (s, 3H), 7.00–7.02 (m, 1H), 7.13–7.18 (m, 2H), 7.28–7.31 (m, 1H), 7.39–7.41 (m, 1H), 7.44–7.46 (m,1H); ^13^C NMR (125 MHz, CDCl_3_): δ = 20.1, 124.7, 126.0, 126.1, 127.1, 127.3, 127.4, 127.7, 127.8, 135.3, 136.4, 139.3, 142.0; IR (neat): ν_max_ = 3105, 2920, 1450, 1231, 826, 696 cm^−1^; HRMS (DART): *m*/*z* calcd for C13H9ClS2 [M + H]+ 264.9912; found: 264.9909.

4-Chloro-2,6-dimethyl-7-(5-methylthiophen-2-yl)benzo[b]thiophene (**6**) 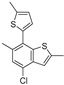


Following the procedure for the preparation of **5**, the reaction using alcohol **4** (65 mg, 0.18 mmol) in 1,2-dichloroethane (20 mL) with SnCl_4_ (1.0 M in dichloromethane, 0.18 mL, 0.18 mmol) in the place of TiCl_4_, gave the crude oil, which was purified by SiO_2_ column chromatography (hexane) to give the desired product **6** (28 mg, 53%).

Colorless oil; Rf = 0.77(hexane); ^1^H NMR (500 MHz, CDCl_3_): δ = 2.32 (s, 3H), 2.52 (s, 3H), 2.55 (s, 3H), 6.74–6.76 (m, 1H), 6.77–6.80 (m, 1H), 6.83–6.85 (m, 1H), 7.17–7.18 (m, 1H); ^13^C NMR (125 MHz, CDCl_3_): δ = 15.3, 16.2, 20.2, 122.5, 125.1, 1225.2, 126.5, 127.2, 127.4, 135.1, 135.9, 137.2, 140.3, 142.0, 142.7; IR (neat): ν_max_ = 3063, 2918, 2857, 1574, 1219, 1001, 802 cm^−1^; HRMS (DART): *m*/*z* calcd for C_15_H_13_ClS_2_ [*M* + H]^+^ 293.0225; found: 293.0223.

4-Chloro-5,6-dimethyl-7-(thiophen-2-yl)benzo[*b*]thiophene (**11**) 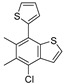


Following the procedure for the preparation of **5**, the reaction using alcohol **10** (666 mg, 2.0 mmol) and TiCl_4_ (1.0 M in 1,2-dichloroethane, 2.0 mL, 2.0 mmol) in 1,2-dichloroethane (100 mL) gave the crude oil, which was purified by SiO_2_ column chromatography (hexane) to give the desired product **11** (266 mg, 48%).

Colorless crystals; Rf = 0.66 (hexane/AcOEt = 30:1); mp 81–82 °C; ^1^H NMR (500 MHz, CDCl_3_): δ = 2.29 (s, 3H), 2.52 (s, 3H), 6.97–6.99 (m, 1H), 7.01–7.04 (m, 1H), 7.14–7.17 (m, 1H), 7.29–7.32 (m, 1H), 7.43–7.45 (m, 1H); ^13^C NMR (125 MHz, CDCl_3_): δ = 17.1, 18.4, 124.8, 125.9, 126.0, 127.0, 127.5, 127.6, 127.7, 131.1, 134.8, 137.0, 139.5, 140.4; IR (neat): ν_max_ = 3017, 2920, 1449, 1323, 1229, 771 cm^−1^; HRMS (DART): *m*/*z* calcd for C_14_H_11_ClS_2_ [*M* + H]^+^ 279.0069; found: 279.0054.

4-Chloro-6-methyl-7-phenylbenzo[*b*]thiophene (**14a**) 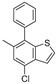


Following the procedure for the preparation of **5**, the reaction using alcohol **13a** (157 mg, 0.5 mmol) and TiCl_4_ (1.0 M in 1,2-dichloroethane, 0.5 mL, 0.5 mmol) in 1,2-dichloroethane (5.0 mL) gave the crude oil, which was purified by SiO_2_ column chromatography (hexane) to give the desired product **14a** (61 mg, 47%).

Pale yellow oil; Rf = 0.55(hexane); ^1^H NMR (500 MHz, CDCl_3_): δ = 2.25 (s, 3H), 6.93–6.96 (m, 1H), 7.27–7.32 (m, 3H), 7.34–7.43 (m, 2H), 7.44–7.52 (m, 2H); ^13^C NMR (125 MHz, CDCl_3_): δ = 19.9, 124.6, 126.2, 126.3, 126.9, 127.3, 128.4 (2C), 129.6 (2C), 133.2, 135.5, 136.4, 139.1, 140.9; IR (neat): ν_max_ = 3057, 2920, 1601, 1442, 1364, 907, 700 cm^−1^; HRMS (DART): *m*/*z* calcd for C_15_H_11_ClS [*M* + H]^+^ 259.0348; found: 259.0361.

4-Chloro-7-(4-chlorophenyl)-6-methylbenzo[*b*]thiophene (**14b**) 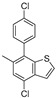


Following the procedure for the preparation of **5**, the reaction using alcohol **13b** (174 mg, 0.5 mmol) and TiCl_4_ (1.0 M in 1,2-dichloroethane, 0.5 mL, 0.5 mmol) in 1,2-dichloroethane (5.0 mL) gave the crude oil, which was purified by SiO_2_ column chromatography (hexane) to give the desired product **14b** (83 mg, 57%).

Colorless oil; Rf = 0.50 (hexane); ^1^H NMR (500 MHz, CDCl_3_): δ = 2.24 (s, 3H), 6.91–6.93 (m, 1H), 7.22–7.25 (m, 2H), 7.29–7.31 (m, 1H), 7.37–7.39 (m, 1H), 7.44–7.47 (m, 2H); ^13^C NMR (125 MHz, CDCl_3_): δ = 19.8, 124.2, 126.2, 127.3, 128.7 (2C), 129.1, 130.6, 131.0 (2C), 133.2, 133.4, 134.1, 137.5, 140.8; IR (neat): ν_max_ = 3103, 2922, 2361, 1558, 1491, 1015, 826 cm^−1^; HRMS (DART): *m*/*z* calcd for C_15_H_10_Cl_2_OS [*M* + H]^+^ 292.9959; found: 292.9937.

6-Methyl-4-phenyl-7-(thiophen-2-yl)benzo[*b*]thiophene (**15**) 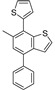


A mixture of **5** (132 mg, 0.50 mmol), PhB(OH)_2_ (91 mg, 0.75 mmol), K_3_PO_4_ (212 mg, 1.00 mmol), Pd(OAc)_2_ (3.4 mg, 0.015 mmol), and SPhos (12 mg, 0.030 mmol) in toluene (1 mL) was stirred at 80–85 °C for 2 h. After cooling down, water was added to the mixture, which was extracted twice with AcOEt. The combined organic phase was washed with water, brine, dried (Na_2_SO_4_) and concentrated. The obtained crude oil was purified by SiO_2_ column chromatography (hexane) to give the desired product **15** (139 mg, 91%).

Colorless crystals; Rf = 0.17 (hexane); mp 136–137 °C; ^1^H NMR (500 MHz, CDCl_3_): δ = 2.42 (s, 3H), 7.06–7.08 (m, 1H), 7.17–7.22 (m, 2H), 7.31–7.33 (m, 1H), 7.37–7.39 (m, 1H), 7.41–7.47 (m, 2H), 7.49–7.54 (m, 2H), 7.74–7.78 (m, 2H); ^13^C NMR (125 MHz, CDCl_3_): δ = 20.2, 124.3, 125.8, 126.7, 126.9, 127.0, 127.5, 128.0, 128.1, 128.2 (2C), 128.8 (2C), 134.3, 136.3, 136.4, 140.2, 140.4, 141.5; IR (neat): ν_max_ = 3028, 2922, 2359, 1576, 1443, 1360, 906 cm^−1^; HRMS (DART): *m*/*z* calcd for C_19_H_14_S_2_ [*M* + H]^+^ 307.0615; found: 307.0600.

4-(4-Methoxyphenyl)-6-methyl-7-(thiophen-2-yl)benzo[*b*]thiophene (**16**) 
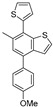


Following the procedure for the preparation of **15**, the reaction of **5** (79 mg, 0.30 mmol) with 4-MeOC_6_H_4_B(OH)_2_ (68 mg, 0.45 mmol), K_3_PO_4_ (127 mg, 0.60 mmol), Pd(OAc)_2_ (2.2 mg, 0.010 mmol), SPhos (8.2 mg, 0.020 mmol) in toluene (1 mL) and the successive purification by SiO_2_ column chromatography (hexane/AcOEt = 30:1) gave the desired product **16** (85 mg, 82%).

Colorless crystals; Rf = 0.44 (hexane/AcOEt = 10:1); mp 105–106 °C; ^1^H NMR (500 MHz, CDCl_3_): δ = 2.41 (s, 3H), 3.89 (s, 3H), 7.03–7.08 (m, 3H), 7.16–7.22 (m, 2H), 7.27–7.30 (m, 1H), 7.36–7.39 (m, 1H), 7.44–7.47 (m, 1H), 7.68–7.71 (m, 2H); ^13^C NMR (125 MHz, CDCl_3_): δ = 20.2, 55.3, 114.2 (2C), 124.3, 125.7, 126.6, 126.7, 127.0, 127.5, 127.7, 129.3 (2C), 132.9, 134.3, 136.1, 136.3, 140.3, 141.4, 159.4; IR (neat): ν_max_ = 2955, 2359, 1611, 1514, 1246, 1179, 906 cm^−1^; HRMS (DART): *m*/*z* calcd for C_20_H_16_O_1_S_2_ [*M* + H]^+^ 337.0721; found: 337.0706.

6-Methyl-4,7-di(thiophen-2-yl)benzo[*b*]thiophene (**17**) 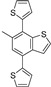


Following the procedure for the preparation of **15**, the reaction of **14a** (79 mg, 0.30 mmol) with 2-thienylboronic acid (58 mg, 0.45 mmol), K_3_PO_4_ (127 mg, 0.60 mmol), Pd(OAc)_2_ (2.2 mg, 0.010 mmol), SPhos (8.2 mg, 0.020 mmol) in toluene (1 mL) and the successive purification by SiO_2_ column chromatography (hexane) gave the desired product **17** (62 mg, 63%).

Colorless crystals; Rf = 0.28(hexane); mp 123–124 °C; ^1^H NMR (500 MHz, CDCl_3_): δ = 2.40 (s, 3H), 7.04–7.06 (m, 1H), 7.16–7.21 (m, 3H), 7.39–7.42 (m, 2H), 7.43–7.47 (m, 1H), 7.49–7.51 (m, 1H), 7.62–7.64 (m, 1H); ^13^C NMR (125 MHz, CDCl_3_): δ = 20.2, 124.4, 125.4, 125.5, 125.8, 126.4, 126.7, 127.0, 127.6, 127.8, 128.5, 129.1, 134.2, 135.1, 140.0, 141.8, 142.3; IR (neat): ν_max_ = 3103, 2922, 2359, 2245, 1576, 1456, 906 cm^−1^; HRMS (DART): *m*/*z* calcd for C_17_H_12_S_3_ [*M* + H]^+^ 312.0101; found: 312.0091.

5,6-Dimethyl-4-phenyl-7-(thiophen-2-yl)benzo[b]thiophene (**18**) 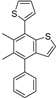


Following the procedure for the preparation of **15**, the reaction of **11** (84 mg, 0.30 mmol) with PhB(OH)_2_ (55 mg, 0.45 mmol), K_3_PO_4_ (127 mg, 0.60 mmol), Pd(OAc)_2_ (2.2 mg, 0.010 mmol), SPhos (8.2 mg, 0.020 mmol) in toluene (1 mL) and successive purification by SiO_2_ column chromatography (hexane) gave the desired product **18** (85 mg, 89%).

Colorless crystals; Rf = 0.29 (hexane); mp 143–144 °C; ^1^H NMR (500 MHz, CDCl_3_): δ = 2.22 (s, 3H), 2.31 (s, 3H), 7.03–7.08 (m, 2H), 7.15–7.19 (m, 1H), 7.20–7.24 (m, 1H), 7.39–7.46 (m, 4H), 7.48–7.54 (m, 2H); ^13^C NMR (125 MHz, CDCl_3_): δ = 17.8, 17.9, 124.2, 125.6, 125.8, 126.9, 127.4, 127.7, 128.1, 128.7 (2C), 129.3 (2C), 131.1, 133.8, 136.0, 138.4, 138.8, 140.6, 141.3; IR (neat): ν_max_ = 3069, 2922, 1601, 1441, 1211, 986, 907 cm^−1^; HRMS (DART): *m*/*z* calcd for C_20_H_16_S_2_ [*M* + H]^+^ 321.0772; found: 321.0778.

5,6-Dimethyl-4,7-di(thiophen-2-yl)benzo[b]thiophene (**19**) 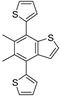


Following the procedure for the preparation of **15**, the reaction of **11** (84 mg, 0.30 mmol) with 2-thienylboronic acid (58 mg, 0.45 mmol), K_3_PO_4_ (127 mg, 0.60 mmol), Pd(OAc)_2_ (2.2 mg, 0.010 mmol), SPhos (8.2 mg, 0.020 mmol) in toluene (1 mL) and successive purification by SiO_2_ column chromatography (hexane) gave the desired product **19** (81 mg, 82%).

Colorless crystals; Rf = 0.29 (hexane); mp 207–208 °C; ^1^H NMR (500 MHz, CDCl_3_): δ = 2.30 (s, 3H), 2.33 (s, 3H), 7.02–7.05 (m, 2H), 7.13–7.15 (m, 1H), 7.16–7.21 (m, 2H), 7.24–7.25 (m, 1H), 7.44–7.46 (m, 1H), 7.47–7.49 (m, 1H); ^13^C NMR (125 MHz, CDCl_3_): δ = 17.9, 18.0, 124.2, 125.7, 126.0, 126.1, 127.0, 127.2, 127.4, 127.5, 128.5, 129.1, 133.3, 133.8, 138.4, 140.4, 140.8, 141.0; IR (neat): ν_max_ = 3103, 2924, 1798, 1 734, 1433, 1366, 1240, 1207 cm^−1^; HRMS (DART): *m*/*z* calcd for C_18_H_14_S_3_ [*M* + H]^+^ 327.0336; found: 327.0337.

6-Methyl-4,7-diphenylbenzo[*b*]thiophene (**20**) 
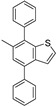


Following the procedure for the preparation of **15**, the reaction of **14a** (104 mg, 0.40 mmol) with PhB(OH)_2_ (73 mg, 0.60 mmol), K_3_PO_4_ (170 mg, 0.80 mmol), Pd(OAc)_2_ (2.7 mg, 0.012 mmol) and SPhos (9.9 mg, 0.024 mmol) in toluene (1 mL), and the successive purification by SiO_2_ column chromatography (hexane) gave the desired product **20** (81 mg, 68%).

Colorless crystals; Rf = 0.36 (hexane); mp 171–172 °C; ^1^H NMR (500 MHz, CDCl_3_): δ = 2.32 (s, 3H), 6.99–7.02 (m, 1H), 7.31–7.34 (m, 2H), 7.35–7.39 (m, 2H), 7.40–7.45 (m, 2H), 7.47–7.54 (m, 4H), 7.76–7.79 (m, 2H); ^13^C NMR (125 MHz, CDCl_3_): δ = 19.9, 124.2, 126.2, 127.0, 127.1, 127.8, 128.2 (2C), 128.3 (2C), 128. 7 (2C), 129.7 (2C), 132.1, 135.4, 135.9, 136.3, 139.9, 140.3, 140.7; IR (neat): ν_max_ = 3053, 2924, 2357, 1599, 1443, 1358, 1213, 1016 cm^−1^; HRMS (DART): *m*/*z* calcd for C_21_H_16_S [*M* + H]^+^ 301.1051; found: 301.1053.

4-(4-Methoxyphenyl)-6-methyl-7-phenylbenzo[*b*]thiophene (**21**) 
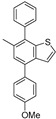


Following the procedure for the preparation of **15**, the reaction of **14a** (78 mg, 0.30 mmol) with 4-MeOC_6_H_4_B(OH)_2_ (68 mg, 0.45 mmol), K_3_PO_4_ (127 mg, 0.60 mmol), Pd(OAc)_2_ (2.2 mg, 0.010 mmol) and SPhos (8.2 mg, 0.020 mmol) in toluene (1 mL), and the successive purification by SiO_2_ column chromatography (hexane/AcOEt = 30:1) gave the desired product **21** (79 mg, 80%).

Colorless crystals; Rf = 0.56 (hexane/AcOEt = 10:1); mp 155–156 °C; ^1^H NMR (500 MHz, CDCl_3_): δ = 2.31 (s, 3H), 3.90 (s, 3H), 6.99–7.01 (m, 1H), 7.04–7.07 (m, 2H), 7.28–7.52 (m, 7H), 7.70–7.73 (m, 2H); ^13^C NMR (125 MHz, CDCl_3_): δ = 19.9, 55.3, 114.1 (2C), 124.2, 126.2, 126.8, 127.1, 128.3 (2C), 129.3 (2C), 129.8 (2C), 132.1, 133.1, 135.1, 135.5, 136.3, 140.0, 140.2, 159.3; IR (neat): ν_max_ = 3034, 2930, 2835, 1609, 1502, 1244, 1034 cm^−1^; HRMS (DART): *m*/*z* calcd for C_22_H_18_OS [*M* + H]^+^ 331.1157; found: 331.1158.

6-Methyl-7-(thiophen-2-yl)benzo[b]thiophen-4-ol (**22**) 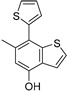


A mixture of **5** (140 mg, 0.53 mmol), Pd(dba)_2_ (5.8 mg, 0.01 mmol), *t*Bu-XPhos (17 mg, 0.04 mmol) and KOH (140 mg, 2.50 mmol) in 1,4-dioxane (0.50 mL) and H_2_O (0.50 mL) was stirred at 100–105 °C for 14 h. After cooling down, 1M HCl aqueous solution was added to the mixture, which was extracted twice with AcOEt. The organic phase was washed with water, brine, dried (Na_2_SO_4_), and concentrated. The obtained crude product was purified by SiO_2_ column chromatography (hexane/AcOEt = 5:1) to give the desired product **22** (105 mg, 80%).

Colorless crystals; mp 114–115 °C; Rf = 0.34 (hexane/AcOEt = 5:1); ^1^H NMR (500 MHz, CDCl_3_): δ = 2.31 (s, 3H), 6.68 (s, 1H), 6.97–6.99 (m, 1H), 7.10–7.12 (m, 1H), 7.13–7.15 (m, 1H), 7.34–7.36 (m, 1H), 7.39–7.42 (m, 1H); ^13^C NMR (125 MHz, CDCl_3_): δ = 20.2, 111.4, 121.8, 124.4, 124.7, 125.5, 126.5, 126.9, 127.5, 135.4, 140.4, 143.0, 149.9; IR (neat): ν_max_ = 3491, 3103, 2959, 2338, 1574, 1352, 1242, 1072 cm^−1^; HRMS (DART): *m*/*z* calcd for C_13_H_10_OS_2_ [*M* + H]^+^ 247.0251; found: 247.0261.

4,4,5,5-Tetramethyl-2-(6-methyl-7-(thiophen-2-yl)benzo[b]thiophen-4-yl)-1,3,2-dioxaborolane (**23**) 
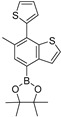


A mixture of **5** (66 mg, 0.25 mmol), bis(pinacolato)diborane (76 mg, 0.30 mmol), NaOAc (31 mg, 0.38 mmol), Pd(dba)_2_ (6.9 mg, 0.012 mmol), and XPhos (11.9 mg, 0.025 mmol) in 1,4-dioxane (0.50 mL) was heated at 95–100 °C for 14 h. After cooling down, water was added to the mixture, which was extracted twice with AcOEt. The combined organic phase was washed with water, brine, dried (Na_2_SO_4_) and concentrated. The obtained crude oil was purified by SiO_2_ (neutral, Kanto Chemical, 60N) column chromatography (hexane/AcOEt = 30:1) to give the desired product **22** (52 mg, 58%).

Pale yellow crystals; mp 93–94 °C; 0.59 (hexane/AcOEt = 10:1); ^1^H NMR (500 MHz, CDCl_3_): δ = 1.42 (s, 12H), 2.37 (s, 3H), 7.02–7.03 (m, 1H), 7.12–7.14 (m, 1H), 7.15–7.17 (m, 1H), 7.38–7.40 (m, 1H), 7.43–7.45 (m, 1H), 7.76 (s, 1H); ^13^C NMR (125 MHz, CDCl_3_): δ = 19.9, 24.9 (4C), 84.3 (2C), 123.2, 125.7, 126.5, 126.9, 127.4 (2C), 132.0, 132.7, 134.3, 140.2, 140.4, 143.3; IR (neat): ν_max_ = 3103, 2976, 2926, 1738, 1580, 1371, 1142 cm^−1^; HRMS (DART): *m*/*z* calcd for C_19_H_21_BO_2_S_2_ [*M* + H]^+^ 357.1158; found: 357.1155.

## 4. Conclusions

We achieved regiocontrolled hetero-type benzannulations of various (2,2-dichlorocyclopropyl)(thiophen-2-yl)methanols to produce uniquely substituted benzothiophenes. The present method involves the distinctive thiophene formation from benzene cores, which is in clear contrast to the traditionally reported methods. 

Furthermore, three types of cross-coupling derivatizations of the obtained stereocongested (less reactive) 4-chlorobenzothiophenes were performed: (i) Suzuki–Miyaura cross-couplings affording various 4-arylbenzothiophenes, (ii) hydroxylation leading to a 4-hydroxybenzothiophene, and (iii) borylation leading to a 4-(pin)B-benzothiophene. This wide variety of hetero-type benzannulations and functionalizations will contribute to synthetic studies, especially for medicinal and material chemistries. 

## Data Availability

Available.

## Supplementary Materials

The following are available online, ^1^H NMR, ^13^C NMR spectra for compounds **3**–**23** (Figure S1–S44), Electronic Supporting Information (S24).

## Sample Availability

Samples of all the compounds **1**–**23** are available from the authors.
